# The predictive Value of Total Neutrophil Count and Neutrophil/Lymphocyte Ratio in Predicting In-hospital Mortality and Complications after STEMI

**DOI:** 10.5681/jcvtr.2014.007

**Published:** 2014-03-21

**Authors:** Samad Ghaffari, Mehdi Nadiri, Leili Pourafkari, Nariman Sepehrvand, Aliakbar Movasagpoor, Neda Rahmatvand, Mohammadamin Rezazadeh Saatloo, Mona Ahmadi, Nader D Nader

**Affiliations:** ^1^Cardiovascular Research Center, Tabriz University of Medical Sciences, Tabriz, Iran; ^2^Tohid Hospital, Sanandaj, Iran; ^3^VA Western New York Healthcare System, University of Buffalo, Buffalo, USA

**Keywords:** Acute Myocardial Infarction, ST Segment Elevation, White Blood Cell, Total Neutrophil Count, Neutrophil/Lymphocyteratio

## Abstract

***Introduction:*** Leukocytosis, predominantly neutrophilia, has previously been described following ST elevation myocardial infarction (STEMI). The exact contribution of this phenomenon to the clinical outcome of STEMI is yet to be shown. We examined cellular inflammatory response to STEMI in the blood and its association with in-hospital mortality and/or adverse clinical events.

***Methods:*** In this cross-sectional study, 404 patients who were admitted with the diagnosis of acute STEMI at Madani Heart Hospital from March 2010 to March 2012 were studied. The complete blood cell count (CBC) was obtained from all patientswithin12-24 hours of the onset of symptoms. Total leukocytes were counted and differential count was obtained for neutrophils, lymphocytes and neutrophil/lymphocyte ratio (NLR) were evaluated. Association of cellular response with the incidence of post-MI mortality/complications was assessed by multiple logistic regression analyses.

***Results:*** In-hospital mortality and post-STEMI complication rate were 3.7% and 43.6%, respectively. Higher age (P=0.04), female gender (0.002), lower ejection fraction (P<0.001) and absolute neutrophil count (P=0.04) were predictors of mortality. Pump failure in the form of acute pulmonary edema or cardiogenic shock occurred in 35 (8.9%) of patients. Higher leukocyte (P<0.03) and neutrophil counts (P<0.03) and higher NLR (P=0.01) were predictors of failure. The frequency of ventricular tachyarrhythmias (VT/VF) at the first day was associated with higher neutrophil count (P<0.001) and higher NLR level (P<0.001). In multivariate analysis neutrophil count was an independent predictor of mortality (OR=2.94; 1.1-8.4, P=0.04), and neutrophil count [OR=1.1, CI (1.01-1.20), P=0.02], female gender [OR=2.34, CI (1.02-4.88), P=0.04] and diabetes [OR=2.52, CI (1.21-5.2), P=0.003] were independent predictors of heart failure.

***Conclusion:*** A single CBC analysis may help to identify STEMI patients at risk for mortality and heart failure, and total neutrophil count is the most valuable in predicting both.

## Introduction

The relationship between inflammation and myocardial infarction (MI) was suggested more than 50 years ago.^1^ Since then, overwhelming evidences supporting this relationship have been obtained from various basic sciences, epidemiological and clinical studies.^2-4^

Determining peripheral leukocyte count is an inexpensive and widely available way to assess the presence of any inflammation. According to the literature, MI is usually accompanied with peripheral leukocytosis^5^ and the leukocytosis is associated with higher rates of short-term mortality and heart failure after myocardial infarction.^6-10^

Neutrophils are the major leukocytes in the peripheral blood. Various clinical trials have reported an association between increased neutrophil count in peripheral blood and short-term post-MI adverse outcomes and worse angiographic findings.^11-13^ Also, there are some reports regarding the value of monocyte count in predicting heart failure following MI.^14,15^

Amongst different hematological indices, it has been shown that the neutrophil/ lymphocyte ratio (NLR) has the highest predictive value in predicting death/MI in high risk patients for coronary artery disease.^16^ Also, it has been shown that NLR predicts the long term mortality in patients hospitalized with ST elevation myocardial infarction (STEMI)^17^, and in patients undergoing percutaneous coronary intervention (PCI).^18,19^

Since neutrophils and lymphocytes act in immunologically different patterns^18^, this study aimed at evaluating the predictive value of total peripheral neutrophil count and NLR in determining the prognosis of MI and the risk of major post-MI adverse events. We hypothesized that patients with high leukocytic response and NLR were at higher risk of in-hospital death and complications after a documented STEMI.

## Materials and methods

In this descriptive cross-sectional study that was approved by the Scientific & Ethical Review Board of Tabriz University of Medical Sciences, four hundred and four patients admitted with acute STEMI in the CCU of Madani Heart Hospital, Tabriz- Iran from March 2010 to March 2012, were enrolled after obtaining an informed written consent. Patients with active inflammation or chronic inflammatory diseases, past history of surgery within 3 months prior to MI, and cancer were excluded from the study. STEMI was determined using the definition and criteria provided by American College of Cardiology (ACC) and European Society of Cardiology; In short: STEMI defined as an increase in cardiac troponin-I (cTNI) along with new ST-segment elevation measured from J point at least 0.2 mV in two adjacent V1-V3 leads or at least 0.1 mV in other leads within 24 hours after the onset of chest pain.

Complete blood cell count (CBC) was performed in all patients within 12-24 hours of onset of symptoms. This was based on the findings of Nunez et al. which showed higher neutrophil and a relatively lower lymphocyte counts resulting in excessive NLR in this time period.^17^ After sampling, blood samples were evaluated for total WBC count, neutrophil, lymphocytes (LYM), and NLR using CBC H1 machine. The information regarding demographic features, past medical history, patient’s outcome and the occurrence of major complications or mortality were collected from the medical records for all subjects.

Hypertension was defined as blood pressure of ≥140/90 mmHg recorded at least two times or current antihypertensive therapy. Diabetes was defined as fasting plasma glucose of>126 mg/dL for at least two measurements or current glucose lowering treatment as defined by the World Health Organization. Hyperlipidemia was defined as total cholesterol of >200 mg/dL or a history of elevated serum total cholesterol during the previous 6 months resulting in prescription of a lipid lowering agent. Primary endpoint was in-hospital mortality and secondary endpoints included pump failure (defined as cardiogenic shock and/or pulmonary edema) and major tachyarrhythmias (defined as atrial fibrillation and ventricular tachycardia or fibrillation).

### Statistical Analysis

All collected data were analyzed using SPSS 18 (SPSS Inc., Chicago, IL). Descriptive statistics were reported as Median (range) for continuous variables and frequency (%) for dichotomous or discrete variables. Chi square test and fisher’s exact test were used for comparing categorical variables. Wilcoxon Rank test was used to compare the numerical data since the data did not have a normal distribution. Multivariate logistic regression was performed to study the role of different factors in predicting adverse post-MI events. Receiver operating characteristics (ROC) curves and Area under the curve (AUC) were used in order to determine the cutoff points for the cell count, and NLR in predicting mortality and major complications. Then, the sensitivity, specificity, positive and negative preventive values, positive and negative likelihood ratio were calculated for each index. Any relationship between WBC subgroups and LVEF were analyzed using Pearson correlation test. P<0.05 was considered statistically significant.

## Results

### Patient characteristics

Four hundred and four patients with acute SETMI were evaluated in this study. 81.2% (328 Cases) of patients were male with the mean age of 58.9±12.9 years. Positive familial history for coronary artery disease was present in 87 (21.5%), hyperlipidemia in 114 (28.2%), Hypertension in 157 (38.9%), DM in 101 (25%), Current smoking in 180 (44.6%) and previous coronary revascularization in the form of either coronary artery bypass grafting (CABG) or percutaneous coronary angioplasty (PTCA) in 12 subjects (3.0%) was detected. The laboratory findings were presented in Table 1 .

**Table 1:** Laboratory findings

**Lab tests**	**Mean±SD**	**Median ** **(95% CI Range)**
Creatine kinase (total)	1,494±90	860 (25-13,700)
Creatine kinase (MB fraction)	153±9	82.5 (3-1,008)
Cardiac Troponin I (cTnI)	10.7±1.2	4.0 (0.01-341)
White Blood Cells count*	11.1±3.6	10.8(2.2-25.8)
Neutrophils absolute count*	8.69±3.59	8.36(0.88-23.29)
Lymphocytes*	1.49±0.74	1.38(0.10-5.27)
Monocytes*	0.65±0.27	0.62 (0.10-2.46)
Neutrophil/Lymphocyte ratio NLR	8.6±10.5	5.7 (0.1-128.4)
Neutrophil/Monocyte ratio NMR	14.9±9.3	12.5 (2.3-115.2)

White Blood Cells, Neutrophils, Lymphocytes and Monocytes were expressed as 1000xcells/mm^3^)

### Electrocardiographic findings

Myocardial infarction was determined to be inferior wall MI in 184 (45.5%) and anterior MI in remaining 220 (54.5%) cases. A total of 176 patients (43.6%) demonstrated electrical complications of MI. Ventricular tachyarrhythmias (VF/VT) were seen in 37 subjects (29 in the first day and 8 cases thereafter), left bundle branch block (LBBB, 14 cases), right bundle branch block (RBBB, 40 cases), atrial fibrillation (20 cases), paroxysmal supraventricular tachycardia (PSVT, 6 cases), and a total of 30 cases of AV block, first degree (20 cases) and second degree (4 cases) and third degree (6 cases).

### Angiographic findings

The frequency of single vessel disease (1VD), 2VD and 3VD was 38.9%, 31.6%, and 29.5%, respectively. According to the angiographic findings, CABG and PCI were suggested respectively for 29.8% and 46.7% of patients and 23.5% of them were candidate for medical follow up.

### Clinical Outcome

Patients were hospitalized for an average of 7.4±4.9 (range 1-35) days. Fifteen patients died during their hospital stay (3.7%). Following complications of STEMI occurred in the order of occurrence; more than moderate MR (29 cases), pulmonary edema (21 cases), cardiogenic shock (19 cases), pericarditis (7 cases), recurrent MI (4 cases) and VSD (2 cases).

### Analysis for post-MI mortality

Non-surviving patients were generally female (*P*=0.04), with advanced age (*P*=0.002), higher cTnI level (*P*<0.001) and lower left ventricular ejection fraction (LVEF) (P<0.001). The mean WBC count and NLR were higher among non-survivors compared to survivors but the differences were not statistically significant. However, absolute neutrophil count was significantly higher among non-survivors ( Table 2). Multivariate analysis revealed neutrophil count as an independent predictor of mortality [OR=2.94, CI (1.03-8.44), *P*=0.04]. Subgroups analysis of WBC by ROC-analysis was performed to determine the sensitivity and specificity of factors in predicting in-hospital mortality. The cutoff point of neutrophil >9.68-x1000 cells/mm3 had a sensitivity of 60% and specificity of 66.2% in predicting post-MI mortality (Figure 1A , AUC=0.65, *P*=0.04).

**Table 2:** Patient variable among the survivors and non-survivors after acute STEM

	**Survivors (n=389)**	**Non-Survivors (n=15)**	***P***
Age (Mean±STD)	58.7±12.9	65.7±13.4	0.04
Gender (Male)	321(82.5 %)	7(46.7 %)	0.002
Hypertension	148(38.1 %)	9(60 %)	0.10
Diabetes mellitus	95(24.4 %)	6(40 %)	0.22
Hyperlipidemia	109(28.1 %)	5(33.3 %)	0.77
Active Smoking	177(45.5 %)	3(20 %)	0.06
Left Ventricular Ejection Fraction (%)	38±10	27±12	<0.001
Creatine kinase (total)	1,482±497	1815±9,157	0.48
Creatine kinase (MB fraction)	152±9	178±44	0.58
Cardiac Troponin I (cTnI)	9.9±0.9	32.9±22.1	<0.001
White Blood Cells count*	10.7 (10.4-11.1)	13.0 (10.1-15.5)	0.10
Neutrophils absolute count *	8.33 (7.82-8.68)	10.99 (7.76-13.06)	0.04**
Lymphocytesabsolute count *	1.38 (1.31-1.44)	1.37 (1.02-1.72)	0.59
Monocytes absolute count *	0.62 (0.58-0.65)	0.61 (0.51-0.91)	0.66
Neutrophil/Lymphocyte ratio (NLR)	5.64 (5.08-6.20)	8.03 (5.66-11.82)	0.28
Neutrophil/Monocyte ratio (NMR)	12.4 (12.1-13.2)	14.4 (10.8-18.9)	0.54

* Median (95% CI; 103×cells/mm^3^); ** Statistically significant on one-tail analysis only.

Figure 1
ROC curves examining various subtypes of peripheral blood leukocytes in predicting in-hospital mortality (A) and complications (B) after STEMI

**1A:**
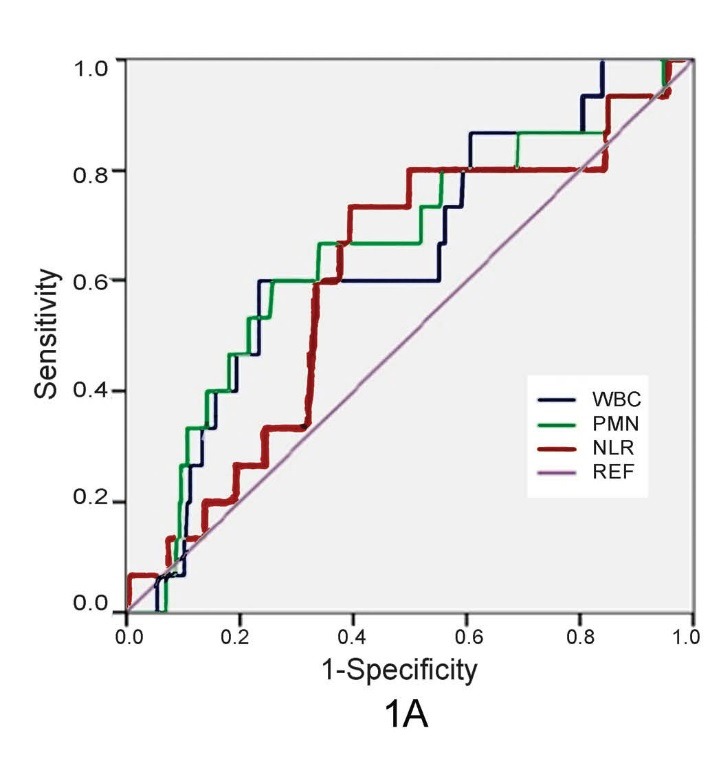

**1B:**
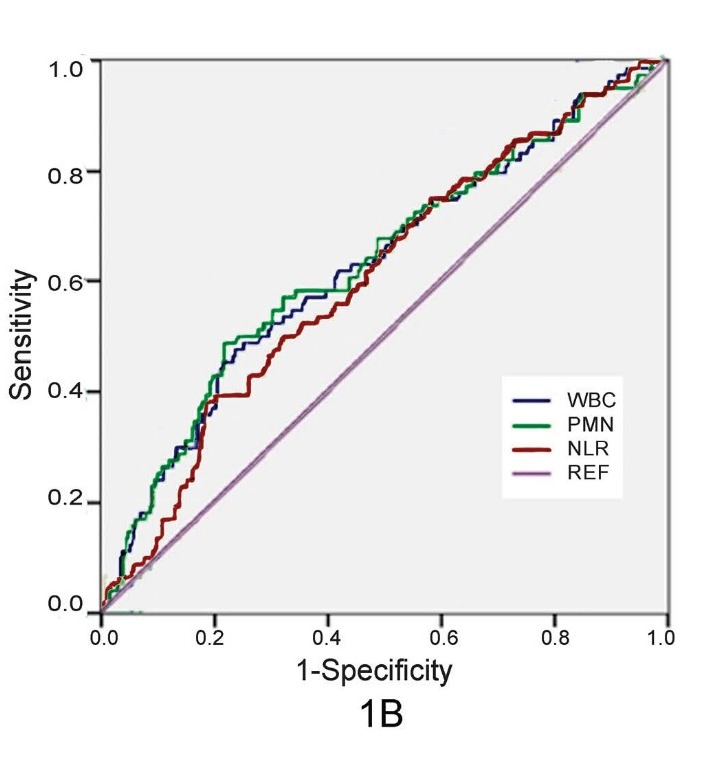

### Analysis for post-STEMI complications

We evaluated the frequency of pump failure and atrial fibrillation or ventricular tachyarrhythmias. Pump failure (defined as cardiogenic shock and/or pulmonary edema) was present in 35 (8.9%) and major tachyarrhythmias (defined as atrial fibrillation, and ventricular tachycardia or fibrillation) in 20 (5.0%) and 37 (9.2%) patients, respectively. Patients with pump failure had significantly higher total WBC (*P*<0.03), neutrophil count (*P*<0.03) and NLR (*P*=0.01). There were no significant differences between total WBC or neutrophil counts and NLR among patients with or without atrial fibrillation and also those with VT/VF beyond first day, however, total WBC count; neutrophil count and NLR were significantly higher in patients with VT/VF within the first day ( Table 3). Multivariate analysis showed neutrophil count, female gender and diabetes as independent predictors for development of pump failure ( Table 4).

**Table 3:** Total WBC and neutrophil counts, and NLR in patients with and without post-STEMI complications

**Complications**	**Parameter**	**Cases with**	**Cases without**	***P***
VT/VF (29) within 24 hours	WBC (1000 cells/mm^3^)	13.2 (11.1-15.6)	10.7 (10.2-11.0)	<0.001
	Neutrophils (1000 cells/mm^3^)	11.37 (8.58-13.44)	8.25 (7.71-8.60)	<0.001
	NLR	11.1 (5.7-13.6)	5.5 (5.0-6.2)	<0.001
VT/VF after 24 hours (8)	WBC (1000 cells/mm^3^)	12.1 (6.7-15.0)	10.7 (10.4-11.1)	0.63
	Neutrophils (1000 cells/mm^3^)	10.41 (3.25-12.78)	8.35 (7.86-8.70)	0.54
	NLR	8.3 (1.8-14.3)	5.7 (5.1-6.2)	0.93
Atrial Fibrillation (20)	WBC (1000 cells/mm^3^)	10.5 (8.7-11.7)	10.8 (10.4-11.1)	0.56
	Neutrophils (1000 cells/mm^3^)	7.85 (6.09-9.86)	8.39 (7.86-8.76)	0.45
	NLR	5.1 (3.9-6.5)	5.7 (5.2-6.4)	0.35
Cardiogenic Shock (20)	WBC (1000 cells/mm^3^)	11.1 (9.9-13.0)	10.7 (10.4-11.1)	0.23
	Neutrophils (1000 cells/mm^3^)	8.58 (7.82-10.99)	8.33 (7.76-8.68)	0.19
	NLR	8.0 (4.2-11.7)	5.7 (5.1-6.2)	0.25
Pulmonary Edema (21)	WBC (1000 cells/mm^3^)	12.8 (11.1-16.4)	10.7 (10.3-11.0)	0.005
	Neutrophils (1000 cells/mm^3^)	10.76 (7.0513.04)	8.30 (7.76-8.61)	0.01
	NLR	8.7 (3.7-12.0)	5.7 (5.1-6.2)	0.41
Pump Failure (35)	WBC (1000 cells/mm^3^)	12.5 (10.7-13.8)	10.7 (10.3-11.0)	0.03
	Neutrophils (1000 cells/mm^3^)	9.65 (8.26-11.30)	8.27 (7.74-8.66)	0.03
	NLR	11.11 (9.4-12.8)	8.0 (7.5-8.5)	0.01

WBC: White Blood Cells; NLR: Neutrophil/Lymphocyte ratio; VT: ventricular tachycardia

**Table 4:** Univariate and multivariate analyses of confounding factors in development of post-STEMI pump failure.

	**With Pump Failure** **(n=35)**	**Without Pump Failure** **(n=369)**	**Univariate** **Analysis**	***P***	**Multivariate** **** **Analysis**	***P***
White Blood Cells	12.5 (10.7-13.8)	10.7 (10.3-11.0)	1.10 (1.02-1.20)	0.01	-	-
Neutrophils	9.7 (8.3-11.3)	8.3 (7.7-8.7)	1.09 (1.01-1.19)	0.03	1.10 (1.01-1.20)	0.02
Diabetes Mellitus	16 (45.7%)	85 (23.0%)	2.81 (1.39-5.71)	0.004	2.52 (1.21-5.2)	0.003
Female/male	12/23	64/305	2.49 (1.18-5.25)	0.02	2.34 (1.02-4.88)	0.04
Prior myocardial revascularization	2 (5.7%)	9 (2.4%)	5.53 (0.98-31.33)	0.05	2.6 (0.50-13.55)	0.25

Univariate analysis was done for the following variables: gender, age, family history, prior myocardial revascularization, prior MI, hypertension, diabetes mellitus, hyperlipidemia, WBC and neutrophil counts and neutrophil-lymphocyte ratio. Five factors were identified as significant confounding variables for pump failure as defined as development of cardiogenic shock and/or pulmonary edema following STEMI. *Since WBC and neutrophil count were collinear variables, we selected only neutrophil count for multivariate analysis.

Similar to mortality, patients with a neutrophil count of >9.68×1000 cells/mm^3^ at the time of admission were found to have significantly higher rate of VT/VF (*P*<0.001) and pulmonary edema (*P*=0.03) during the first 24 hours compared to those who had a neutrophil count of less than or equal to 9.68×1000 cells/mm^3^ ( Table 5).

**Table 5:** Post-STEMI complications in two groups with high and low neutrophil levels

	**Neutrophil >9.68** **(n=140)**	**Neutrophil ≤9.68** **(n=264)**	***P***
VT/VF within 24 hours (Early)	19 (13.6%)	10 (3.8%)	<0.001
VT/VF after the first day (Delayed)	4 (2.9%)	4 (1.5%)	0.45
Atrial Fibrillation	6 (4.3%)	14 (5.3%)	0.81
Cardiogenic shock	8 (5.7%)	11 (4.2%)	0.47
Pulmonary edema	12 (8.6%)	9 (3.4%)	0.003
Pericarditis	3 (2.1%)	1 (0.4%)	0.12

Different WBC indices (WBC and neutrophil count & NLR) were analyzed by ROC analyses in order to determine the sensitivity and specificity of factors in predicting in-hospital post-MI complications. AUC were significant for all of three leukocytic indices of inflammation, but neutrophil count had more sensitivity than other indices (AUC=0.628, P<0.001, Figure 1B).

## Discussion

In the current study, we evaluated the leukocytic response to STEMI and examined its possible association with in-hospital mortality and post-infarction complications. We demonstrated that 12-24 hours following STEMI the numbers of white blood cells, mostly in the form of neutrophils, are higher than known normal values. Increased neutrophil count was associated with higher in-hospital mortality, post-infarction pump failure and occurrence of serious ventricular arrhythmias within the first 24 hours. The presence of neutrophilia after STEMI (higher than the cutoff value of 9.68×1000 cells/mm3) was predictive of pump failure and significant increase in the frequency of ventricular arrhythmias within the first post MI day. Comparably, higher NLR was also associated with higher frequency of acute pump failure and first day arrhythmia following STEMI.

Similar reports by Menonet al., suggested that patients with higher leukocyte count were at high risk of heart failure and cardiogenic shock.^9^ Barron et al. demonstrated that there was an association between high leukocyte count and incidence of cardiogenic shock or congestive heart failure.^10^ They reported a higher mortality in patients with more intense increase in WBC count. A similar linear correlation was observed between in-hospital mortality and increasing white blood cells count in the blood.^8^ In our study, although there was a higher WBC count among non-survivors, the difference was not statistically significant. We postulated that lower in-hospital mortality rate in our patient population was accountable for this finding.

We performed a single CBC analysis to show the value of this inexpensive and widely available test in risk stratification post-STEMI complications. This was based on the findings of Nunez et al. that showed highest neutrophil and lowest lymphocyte counts and maximum NLR in 12-24 hours following STEMI had a higher overall long-term mortality.^17^ However, we found that the NLR was inferior to the absolute number of the neutrophils in predicting in-hospital mortality.

The frequencies of pulmonary edema and VF/VT within the first day of admission were associated with higher levels of neutrophils and NLR. Association between higher neutrophil count and heart failure has been stressed in several studies.^20,21^ Chia et al. showed that elevated leukocyte and neutrophil counts after primary PCI in patients with STEMI were associated with larger myocardial infarct size and lower LVEF and were independent predictors of cardiovascular outcome.^22^ Similarly, our study patients with evidence of heart failure either as pulmonary edema or cardiogenic shock had higher WBC count, neutrophil and NLR ratio.

The association between inflammation and atrial fibrillation is well studied.^23^ In our study there was no significant difference in total WBC, neutrophils or NLR between those with and without AF. We did not use routine holter monitoring in our patients and detailed arrhythmia evaluation was not possible after second or third day when patients were transferred from CCU and this may be a confounding factor for this analysis. Data regarding association of ventricular arrhythmias with CBC results are conflicting.^18^ Chatterjee et al. reported that pre-procedural elevated WBC count, neutrophilia and elevated NLR in patients undergoing PCI were significant predictors of ventricular arrhythmias.^24^ In our study, higher WBC, neutrophil count and NLR were found in patients who developed VT/VF within the first day but the difference was not significant for arrhythmias beyond this time course. We postulated this might be secondary to the stress associated with cardioversion and possible cardiopulmonary resuscitation in these patients rather than a primary causative factor.

In our study NLR did not have a significant relationship with mortality but was found to have a direct relationship with frequency of heart failure and development of ventricular arrhythmias within the first day. In the studies of Nunez et al. NLR level was an independent predictor of in-hospital mortality.^17,25^ In addition, Horne et al. found that neutrophil, lymphocyte and NLR were independent factors for predicting death/MI, however among them, NLR was more powerful predictor of the risk of death/MI.^16^ We believe that lower mortality rate in our study may be responsible for this finding.

In summary, our study showed that this simple and widely available test might help to identify STEMI patients who are at a higher risk of death and developing heart failure and may help in risk stratification of these cases. Higher neutrophil count was found to have the best predictive value for both mortality and heart failure.

## Ethical issues

All patients gave written informed consents and the study

was approved by our local Ethics Committee.

## Competing interests

The authors of the present work declare that there is no

conflict of interest.
